# Design of Mobile Augmented Reality in Health Care Education: A Theory-Driven Framework

**DOI:** 10.2196/mededu.4443

**Published:** 2015-09-18

**Authors:** Egui Zhu, Anneliese Lilienthal, Lauren Aquino Shluzas, Italo Masiello, Nabil Zary

**Affiliations:** ^1^ Centre for Learning and Knowledge Department of Learning, Informatics, Management and Ethics Karolinska Institutet Stockholm Sweden; ^2^ Center for Design Research Stanford University Stanford, CA United States; ^3^ Mohammed VI University of Health Sciences Casablanca Morocco

**Keywords:** augmented reality, health care education, antibiotics, general practitioners, learning environment, learning theory, mobile technology

## Abstract

**Background:**

Augmented reality (AR) is increasingly used across a range of subject areas in health care education as health care settings partner to bridge the gap between knowledge and practice. As the first contact with patients, general practitioners (GPs) are important in the battle against a global health threat, the spread of antibiotic resistance. AR has potential as a practical tool for GPs to combine learning and practice in the rational use of antibiotics.

**Objective:**

This paper was driven by learning theory to develop a mobile augmented reality education (MARE) design framework. The primary goal of the framework is to guide the development of AR educational apps. This study focuses on (1) identifying suitable learning theories for guiding the design of AR education apps, (2) integrating learning outcomes and learning theories to support health care education through AR, and (3) applying the design framework in the context of improving GPs’ rational use of antibiotics.

**Methods:**

The design framework was first constructed with the conceptual framework analysis method. Data were collected from multidisciplinary publications and reference materials and were analyzed with directed content analysis to identify key concepts and their relationships. Then the design framework was applied to a health care educational challenge.

**Results:**

The proposed MARE framework consists of three hierarchical layers: the foundation, function, and outcome layers. Three learning theories—situated, experiential, and transformative learning—provide foundational support based on differing views of the relationships among learning, practice, and the environment. The function layer depends upon the learners’ personal paradigms and indicates how health care learning could be achieved with MARE. The outcome layer analyzes different learning abilities, from knowledge to the practice level, to clarify learning objectives and expectations and to avoid teaching pitched at the wrong level. Suggestions for learning activities and the requirements of the learning environment form the foundation for AR to fill the gap between learning outcomes and medical learners’ personal paradigms. With the design framework, the expected rational use of antibiotics by GPs is described and is easy to execute and evaluate. The comparison of specific expected abilities with the GP personal paradigm helps solidify the GP practical learning objectives and helps design the learning environment and activities. The learning environment and activities were supported by learning theories.

**Conclusions:**

This paper describes a framework for guiding the design, development, and application of mobile AR for medical education in the health care setting. The framework is theory driven with an understanding of the characteristics of AR and specific medical disciplines toward helping medical education improve professional development from knowledge to practice. Future research will use the framework as a guide for developing AR apps in practice to validate and improve the design framework.

##  Introduction

Augmented reality (AR) is a leading topic in media consumption, education, health care, commerce, security and a range of areas involving the development of mobile technologies, such as wearable devices, cloud computing, mobile phones, and tablets. AR was coined to describe a worker-training app in which a computer-produced diagram is superimposed and stabilized in a specific position on a real-world object [1]. AR is defined as a real-time direct or indirect view of a physical real-world environment that is enhanced or augmented by adding virtual computer-generated information to it [2]; Carmigniani and Furht’s work focused on AR that is interactive and registered in 3D. The International Organization for Standardization (ISO), an international organization that develops and publishes international standards for audio and video coding, defines AR as a live view of a real-world environment whose elements are augmented by computer-generated content, such as sound or graphics [3]. This definition refers to any computer-generated content that can be used to enhance the real physical environment.

Education frequently intersects with the AR evolution because AR has the following characteristics:

1. AR provides users with an authentic and situated experience, when connected with the surrounding real-world environment.
2. AR enhances the physical environment around users with virtual information that becomes interactive and digital.
3. AR shows users an indirect view of their surroundings and enhances users’ senses through virtual information.

When companies were developing early versions of AR, an important focus area was workplace training.

Within health care education, AR has been used across a range of subject areas. In our preintegrative review of papers published before November 2012 [4], we identified 2529 research papers in the Education Resources Information Center (ERIC), the Cumulative Index to Nursing and Allied Health Literature (CINAHL), MEDLINE, Web of Science, PubMed, and SpringerLink through computerized searching with two groups of words: *augmented reality* and its synonyms, and *medical education* and its synonyms. A total of 439 full papers were checked and 77 matched the content criteria. We analyzed 25 of the papers that clearly described a research question and/or aim, research results, data collection, and analysis processes. The results showed that AR is useful for health care learning, and that learners accepted AR as a learning technology. The acceptance of AR was verified by our preliminary interviews with two managers and three physicians in two community hospitals in China.

In our preintegrative review, most papers claimed that AR is beneficial for health care learning. Specific benefits included the following: reducing the amount of practice needed, reducing failure rate, improving performance accuracy, accelerating learning speed and shortening learning curves, capturing learners' attention, improving one’s understanding of spatial relationships, providing experiences with new types of authentic science inquiry, and improving the assessment of trainees. However, few papers mentioned using learning theory to guide the design or application of AR for health care education. Instead, the traditional learning strategy, “see one, do one, and teach one,” was used to apply the new technology.

A design framework connects concepts with applied problems in order to provide a comprehensive understanding of a phenomenon and to guide practice [5]. An instructional-design framework that supports goals, values, and systematic methods has been shown to overcome the shortcomings of a technology-driven approach, which traditionally has been used to design technology-enhanced training programs [6]. However, in our comprehensive literature search, we did not find a published design framework that guides the design and development of AR in health care education.

The spread of antibiotic resistance has become a major threat to global public health [7]. A health systems perspective was suggested to solve the dangers and ethical dilemmas of current use, misuse, and overuse of antibiotics [8]. General practitioners (GPs) are an essential part of medical care throughout the world, and their education in rational antibiotic use should enhance care in higher-income and lower-income settings [9]. Evidence shows that the effects of GP training in appropriate antibiotic use varies [10]. Well-designed medical education has been shown to improve targeted antibiotic prescribing outcomes [11]. However, evidence also shows that educational outreach often fails in more experimental settings due to insufficient workability where the education does not “fit” with the work environment [12]. In addition, drug-centered pharmacology teaching or disease-centered diagnostic clinical training has been weak in transforming pharmacological knowledge into clinical practice [13]. To address this health care education challenge, our study examined the use of augmented reality as a powerful partner to bridge the gap between knowledge and practice.

Mobile technology, which is portable and can be easily immersed in different environments, is developing rapidly. According to a report by Morgan Stanley, by 2020 the use of mobile Internet computing is projected to surpass desktop Internet usage by over 10 times [14]. There are currently more than 100,000 health care apps available [15], and current mobile tools—tablets, mobile phones, and other wearable devices—include features that rival existing AR tools (eg, built-in video cameras, global positioning systems [GPS], wireless receivers, and sensors) [16]. This integration of embedded devices can facilitate the ability to track learners in their natural environment and objects that enhance learning [17]. In health education, app-based mobile devices have been shown to support individual and social aspects of learning [18]. The benefit of mobile phone use in health care has also been shown for evaluating interventions with antibiotic treatment [19].

In short, AR with mobile technology has the potential to transform health education, yet lacks an effective framework for guiding the design, development, and application of such tools. AR can change the effects of GP training in the appropriate use of antibiotics, in an effort to reduce threats from existing global health epidemics.

This study aimed to develop a mobile augmented reality education (MARE) design framework that would guide the development of AR educational apps for health care settings. We used the rational use of antibiotics as a context for piloting MARE. This study addresses the following research questions:

1. What learning theories are suitable for guiding the design of an AR education app?
2. What factors should be involved in designing the MARE framework to support effective health care education through AR?
3. How can the developed design framework be applied in the context of a health educational challenge, such as improved prescribing of antibiotics?

## Methods

### Overview

Translating new information into clinical practice depends on six types of systems, each with its own purpose and agenda [20]. These systems include the health care environment, the physician him/herself, relevant clinical information, continuing medical education, implementation of a clinical strategy, and clinical regulatory oversight. The multidisciplinary views provided by such systems could be useful in designing, developing, and applying AR in health care education.

### Development of the Mobile Augmented Reality Education Design Framework

The design framework for MARE was built using a conceptual framework analysis method (CFAM) [5]. CFAMs, based on grounded theory qualitative methods, are multidisciplinary research approaches aimed at invoking critical thinking during the iterative processes of the research [21]. CFAMs are used to generate conceptual frameworks from multidisciplinary publications and reference materials. These frameworks connect the problems identified with the concepts to be applied in order to provide understanding of a phenomenon [5]. CFAMs have been applied in designing conceptual frameworks that illustrate social considerations for information technology in health care, education, and work/practice research [21-23]. The CFAM’s multidisciplinary approach makes this analysis method particularly suitable for designing a MARE framework, since learning is a complex process.

Jabareen suggested that a CFAM is composed of eight steps:

...a) mapping selected data sources; b) reviewing the literature and categorizing the selected data; c) identifying and naming the concepts; d) deconstructing and categorizing the concepts; e) integrating the concepts; f) synthesis, re-synthesis, and making it all make sense; g) validating the conceptual framework; and h) rethinking the conceptual framework. [24]

To design the framework, we collected data from research papers, government reports, conference papers, and websites, as well as documentation of instructional experiences across areas such as medicine, public health, education, instructional design, information technology, and management. Directed content analysis was used to analyze the collected data. This analysis was guided by a structured process and was particularly useful for conceptually developing a theoretical framework [25]. The initial coding of categories starts with instructional system design theory, which involves following the principle of instructional design to promote effective, efficient, and engaging instruction by asking what, how, and why [26]. The study’s lead author (EZ) used direct content analysis to identify key concepts and determine how they might be related within a framework. The concepts were then discussed with the study’s principal investigator (NZ). EZ created the framework, as well as the supporting figures to aid future instructional designers in use of the framework, and piloted the framework in collaboration with members of the research team. The framework and supporting figures were then discussed among the authors and resynthesized to support the aims of the study and to improve future usability of the framework by readers.

### Application of the Mobile Augmented Reality Education Design Framework to an Educational Challenge

The MARE design framework was applied in designing AR for GPs’ rational use of antibiotics education. This application of MARE and a subsequent AR program could help solve a major health care educational challenge. The framework was also a step toward the validation of MARE through its application.

First, following our development of the MARE design framework, a systemic architecture, which was provided by the main framework, helped handle the main factors of the application of MARE to GPs’ rational use of antibiotics. Second, the data—specific to education on rational use of antibiotics by GPs—were acquired and analyzed. We collected learner abilities and the rational therapeutic process from report authorities such as the World Health Organization and Public Health England, and then examined the results of rational use within medical and health education. Next we used expected learner abilities to describe the learning outcomes and analyzed the GP learners’ personal paradigm with the rational therapeutic process. Last, we compared the learning outcomes and the GP personal paradigm, and used the MARE function structure to define learning environments and design learning activities that would be useful for GPs to improve their ability and develop their own paradigm for the rational use of antibiotics. The learning environment and learning activity design were guided by the learning theories.

## Results

### The Mobile Augmented Reality Education Design Framework

#### Overview

**Figure 1:**
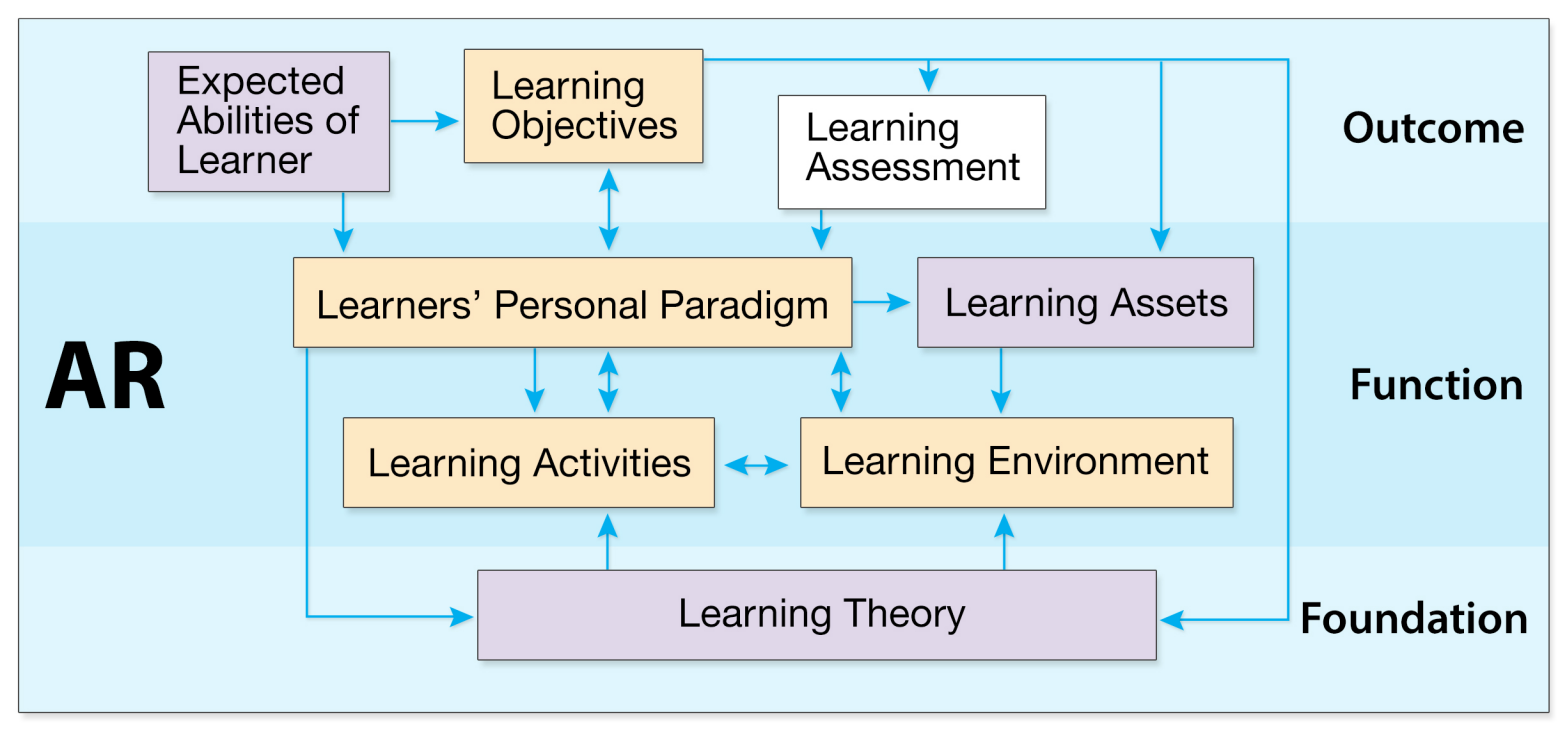
The main elements of the MARE design framework.

The relationships among the key concepts we identified using the CFAM informed the following framework shown in Figure 1. The learner is central to the instructional design guided by this MARE framework. These concepts include learning theories, objectives, assessment, activities, environment, materials, and the personal paradigm. They have been mapped to three main layers of MARE—foundation, outcomes, and function.

The three main layers of the framework provide the hierarchical structure for the content objects. The design order started with defining learning objectives in the outcome layer. We then developed the foundation through examining theories that support the MARE framework and its associated AR characteristics. Finally, we focused on designing the function level, which was guided by the learning theories, in an effort to achieve the outcomes.

The relationships between the layers and concepts are illustrated in the following figure through the use of arrows and colors. As the framework design was an iterative process, the AR function layer is the design object, while the foundation and outcome layers provide support to achieve the design aim. The factors within a layer (colored orange and purple) should be considered while designing each layer. The four key elements shown in orange are highlighted in the framework. The purple factors help to support each layer, as needed. One-way arrows pointing to a concept are influenced by their starting ideas. The two-way arrows align with the concepts, as both the source and the target of relationships.

#### Foundation Layer

The foundation provides the reasons why MARE is useful for health care education and considers our first question regarding which learning theories are suitable. Different learning theories provide different views on learning. Learning theory is the foundation for devising learning activities, organizing study content and materials, and establishing learning environments. Guided by suitable learning theory, AR can perform optimally in health care education [27].

#### Function Layer

Function tells us how health care learning could be achieved with MARE. The function depends upon the learners’ personal paradigms, which we will define and discuss more deeply below, and provides support for the outcome and foundation levels. Learning requires suitable material and activities in an appropriate environment. These learning materials and activities should be selected and developed by considering the learning objectives and the learners’ paradigm, along with the AR learning environment. The choice of activities and environments should be grounded in learning theory from the foundation level and the characteristics of AR.

#### Outcome Layer

The outcome helps us understand which abilities health care learners may achieve through MARE and informs how to design the functional level of MARE. Professional certification requirements and the learner’s paradigm include preknowledge and influence the learning objectives. Meanwhile, the learning assessment standards, as part of the outcome level, should be ascertained according to the specific learning objectives.

### The Outcome Layer Combined With Miller’s Pyramid and Bloom’s Taxonomy

#### Overview

First, we consider the outcomes for MARE, which are concerned with the learner’s abilities. We combined certification criteria with learning objectives and assessment measures based upon well-established educational frameworks. Miller’s pyramid of clinical assessment provides understanding of transforming knowledge to action in medical education [28]. However, most of this research focuses on action verbs adapted from Miller’s pyramid [29]. Simply using action verbs on behalf of low cognition levels as the entire ability on Bloom’s taxonomy leads to "teaching pitched at the wrong level" [30].

**Figure 2:**
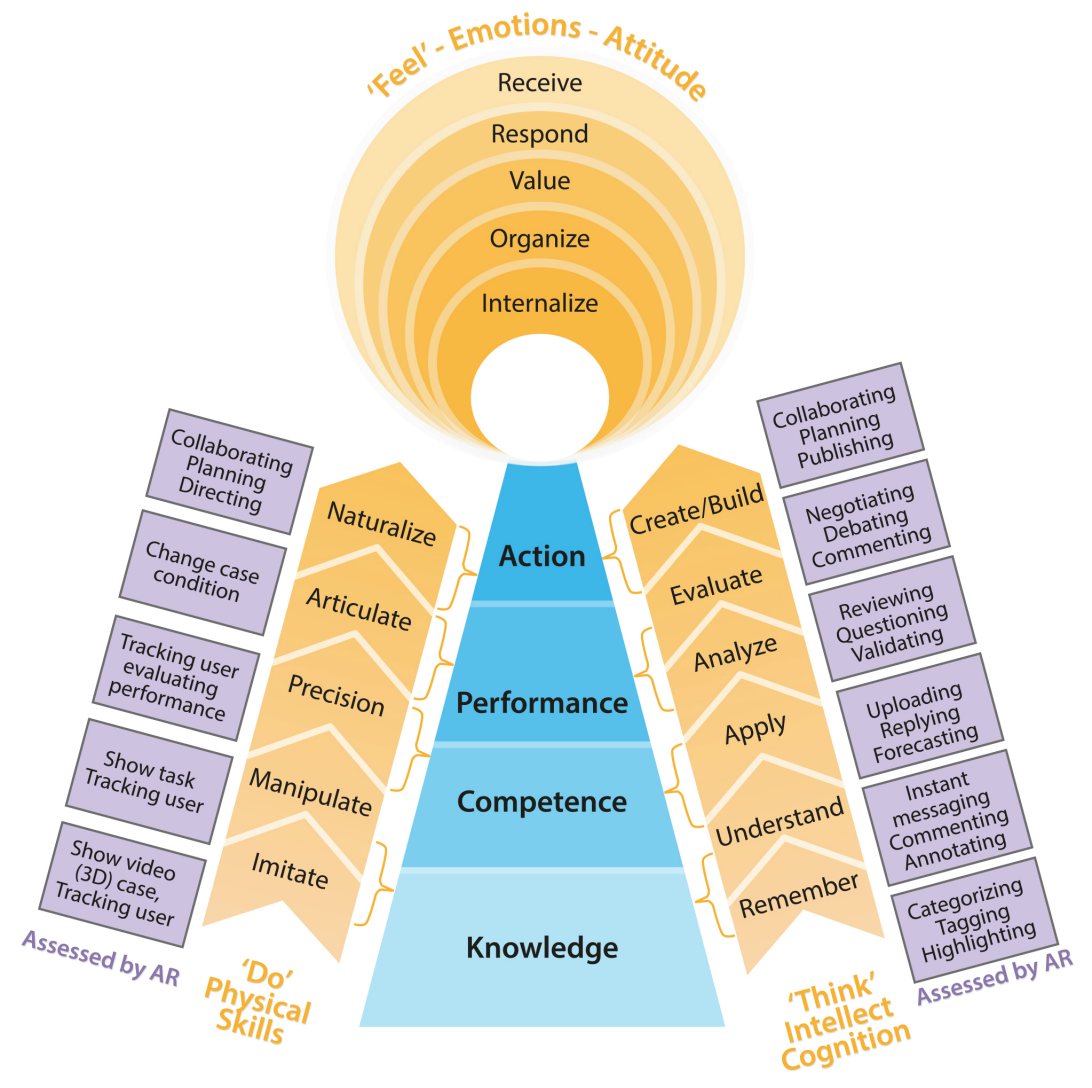
Ability frames from knowledge to action: how to evaluate MARE outcomes.

Bloom’s taxonomy and its development have been used for planning, designing, assessing, and evaluating training and learning effectiveness around the world. Three domains—the cognitive domain, the psychomotor domain, and the affective domain—have each been ordered by the degree of difficulty. The three domains, also known as cognition, skill, and attitude, are independent but influence one another. Figure 2 shows the integrated hierarchy of an ability model and how to evaluate MARE outcomes, from knowledge to action. Anderson’s adapting cognitive domain, Bloom’s affective domain, and Dave’s psychomotor domain were adapted for MARE [31-33]. As we move from knowledge to action, we see different ability levels form different cognitive and physical skills. Only the affective domain did not map directly to ability level, but affected ability achievement.

#### Knowledge Level

Knowledge is about knowing facts, information, descriptions, or skills. Knowledge includes procedural knowledge and declarative knowledge. Procedural knowledge, which is the skill within the knowledge level (KS), can be evaluated by imitating or manipulating. Declarative knowledge, which is the cognition within the knowledge level (KC), can be tested by remembering or understanding. Although attitude is not part of knowledge, attitude affects health care student learning knowledge. Knowledge can be assessed by tracking the students’ behaviors during the health care learning process in MARE.

#### Competence Level

Competence is the ability to apply knowledge or a precise behavior in the practical context. Competence is about knowing and doing something the right way. Emotions and values not only affect the application of knowledge, but are also a foundation upon which to build competence according to physicians’ professional competence definitions [34]. The cognition within the competency level (CC) can be evaluated by reliably solving problems, and the skill within the competency level (CS) can track and test physicians operating in real circumstances or simulated practice.

#### Performance Level

Performance is the quality needed to accomplish work, acts, and achievements in real clinical practice. Performance is based on competence, but is influenced by government, organizations, patients, and individual physician factors [35]. Performance requires higher cognitive ability, such as analyzing and evaluating. A real case from the physician clinical setting is provided for the physicians to analyze and evaluate in MARE. Thus, physicians will analyze and evaluate their cognition within the performance level (PC). Higher performance is articulated in the skills demonstrated. The skill within the performance level (PS) can be tracked and evaluated. Since personal performance is affected by many other factors, improving performance cannot be separated from organizing attitudes or values, which requires comparing and synthesizing different values to resolve conflicts.

#### Action Level

Action is the ability to run a series of events for a given set of processes in health care to optimize patient outcomes. The results of action come from one’s individual performance as well as collaboration with other colleagues and shared decision making with patients, caregivers, or advocates where appropriate. At the action level, the skill is naturalizing behaviors, and the core of cognition is focused upon creating new meaning or structure. The skill within the action level (AS) and the cognition within the action level (AC) can be evaluated through patient outcomes and the impact on other physicians.

By applying this ability frame from knowledge to action, the expected abilities of GPs’ rational use of antibiotics are specifically described, as shown in Tables 1-4. Every item that is a component of the expected abilities uses the verbs from Bloom's taxonomy to make the item easy to execute and evaluate. We can use the related assessed ways in Figure 2 to design how to evaluate them in MARE.

**Table 1:** Expected general practitioner knowledge of rational use of antibiotics.^a^

Domain	Expected knowledge
Cognition	Stating public health antibiotics national guidelines Recognizing trade and generic names, and the class of prescribed antimicrobial Understanding the nature and classification of pathogenic microorganisms Understanding the principles of prevention, treatment, and control of infection Understanding the modes of action of antibiotics: broad versus narrow spectrum Understanding the mechanisms of antimicrobial resistance Understanding local microbial-/antimicrobial-susceptibility patterns Understanding of common side effects, including allergy, drug/food interactions, and contraindications of the main classes of antimicrobials Interpreting basic microbiological investigations Interpreting clinical and laboratory biological markers
Skills	Obtaining microbiological cultures or other relevant tests before commencing treatment as necessary Implementing microbiological and other investigations to diagnose and monitor the response to treatment of infections and their complications Choosing in case of prior use of antibiotics when selecting an antibiotic for empiric therapy
Attitude	Understanding the importance of taking microbiological samples for culture before starting antibiotic therapy Understanding the importance of monitoring for common side effects, including allergy, drug/food interactions, and contraindications of the main classes of antimicrobials Responding to the importance of selection advantages

^a^The table content was developed using various sources [36-38].

**Table 2:** Expected general practitioner competencies for rational use of antibiotics.^a^

Domain	Expected competencies
Cognition	Selecting and prescribing antibiotic therapy according to national/local practice guidelines Using local microbial-/antimicrobial-susceptibility patterns when conducting empiric treatments Using antimicrobial agents for prophylaxis appropriately Constructing the prescription for an antimicrobial with its pharmacokinetics and knowing how this affects the choice of dosage regimen
Skills	Choosing and calculating the dose, route, and interval of administration Monitoring the therapeutic drug and adjusting doses to ensure adequate drug levels Using the antibiotics toolkit
Attitude	Not initiating antibiotic treatment in the absence of bacterial infection Avoiding the unnecessary use of broad-spectrum antimicrobials Using only single doses of antimicrobials for surgical and other procedures for which prophylaxis has been shown to be effective unless published national recommendations suggest otherwise

^a^The table content was developed using various sources [36-38].

**Table 3:** Expected general practitioner performances with rational use of antibiotics.^a^

Domain	Expected performances
Cognition	Applying best bacteriological guess for empiric therapy Estimating the shortest possible adequate duration Assessing when not to prescribe antimicrobials, and use of alternatives Reassessing the antibiotic prescription around day 3
Skills	Switching to the correct antimicrobial based on microbiological results and cost effectiveness Mastering delayed antimicrobial prescription and negotiation with the patient Inputting documentation in the prescription chart and/or in patients’ clinical records
Attitude	Working within ethical code of conduct Applying legal and ethical frameworks affecting antibiotic-prescribing practice

^a^The table content was developed using various sources [36-38].

**Table 4:** Expected general practitioner actions with rational use of antibiotics.^a^

Domain	Expected actions
Cognition	Engaging the views of others and cooperating with others with more expertise in antimicrobial treatment policy decisions Educating patients and their caregivers, nurses, and other supporting clinical staff Engaging regularly in team-based measurement of the quality and quantity of antimicrobial use Sharing with prescribers, as well as informing antimicrobial surveillance/infection prevention and control measures
Skills	Using the results of adverse-event monitoring, laboratory susceptibility reports, antimicrobial prescribing audits, and antimicrobial usage data Producing sustained improvements in the quality of patient care Using locally agreed-upon process measures of quality, outcome, and balancing measures
Attitude	Adapting consultations and prescribing to meet patient diversity Ensuring that confidence and competence to prescribe are maintained Maintaining patient confidentiality, dignity, and respect in line with best practice, regulatory standards, and contractual requirements

^a^The table content was developed using various sources [36-38].

### The Learning Theories Supporting the Foundation Layer

Understanding learning theories and their interpretations can boost the use of effective teaching and learning strategies for medical education practice [27]. However, as we emphasized, few AR programs in health care education use learning theory to guide the design, development, and application. AR has the potential to provide powerful contextual situated learning experiences and to aid in exploring the connected nature of information in the real world. According to the requirement for transforming health care education and these characteristics of AR, we selected three learning theories guiding MARE design: situated learning, experiential learning, and transformative learning. Table 5 shows the main characteristics of these learning theories and how they can inform MARE design.

The reasons we chose these three learning theories are as follows:

1. From the view of social practice, situated learning theory provides a holistic perspective on the interpretation of learning by exploring the situated characteristics of the learners, environment, and practice [39].
2. Experiential learning theory emphasizes the dynamic state between the learner and the environment [40]. This theory differs from situated learning theory by focusing more on the experiences of an individual learner to create knowledge.
3. Transformative learning focuses on discovering evidence that education facilitates changes in the learner’s frames of reference or schema by which the learner identifies his or her life world [41].

These three learning theories provide different views of learning and enhance different characteristics of learning environments, which AR could provide. In addition, the learning activities suggested by these theories could be applied in MARE. Each learning theory can inform decisions for the learning environment, activities, and how to apply AR.

Learning environments that encompass the physical, social, and psychological context of learning are of significant importance and require the attention of medical and other health sciences educators when they teach or design a course [44]. The medical learning environment could be in medical school, an academic health center, or a clinical environment. Different learning settings are supported by different types of learning environments.

**Table 5:** Comparison of situated learning, experiential learning, and transformative learning.^a^

Characteristics	Situated learning	Experiential learning	Transformative learning
Learning assumption	A dimension of social practice	A holistic process of adapting to the world	Critically aware of the personal paradigm
Learning perspective	Concerns the whole person acting in the world	Combines experience, perception, cognition, and behavior	Implicates transformation in meaning perspective that encompasses cognitive, conative, and affective components
Definition	Learning is participation in communities of practice, which produces knowledgeable identities and the community itself	Learning is the processing of transformative experiences, which includes concrete experience and abstract conceptualization	Learning is changing problematic frames of reference, which comprise habits of mind, points of views, and mind-sets
Environmental conditions	Real-life situation where the learning occurred	Create learning environments for feeling and thinking, reflecting, and acting	Safe environment, authentic settings
Learning activities	Sustained participation via observation, collaboration, and communication	Reflective observation and active experimentation	Critical reflection and dialectical discourse to validate beliefs, intentions, values, and feelings
Implications for MARE^b^	Thinking holistically about learning activities, real practical tasks, real environments, and MARE functions Understanding and using the social situation, especially the real-life environment Design activities to sustain participation with MARE (or AR^c^)	Design virtual learning environment for feeling, thinking, watching, and doing Utilize Kolb’s spiral model from concrete experience or knowledge to action [40]	Design the learning activities to reflect upon and change problematic frames of reference

^a^The table content was developed using various sources [39-43].

^b^Mobile augmented reality education (MARE).

^c^Augmented reality (AR).

### The Functional Level Design

#### Overview

MARE provides a prompt, portable tool for medical student learning within the clinical setting in order to transform knowledge into practice. The flexible personal paradigm, which is “more inclusive, discriminating, open, reflective, and emotionally able to change,” is more appropriate for guiding action [43]. The most important function of AR is mixing aspects of the real environment with virtual objects to create different learning environments. As backed by the learning theories previously discussed, these mixed environments will be useful for the medical student to form a flexible personal paradigm.

**Figure 3:**
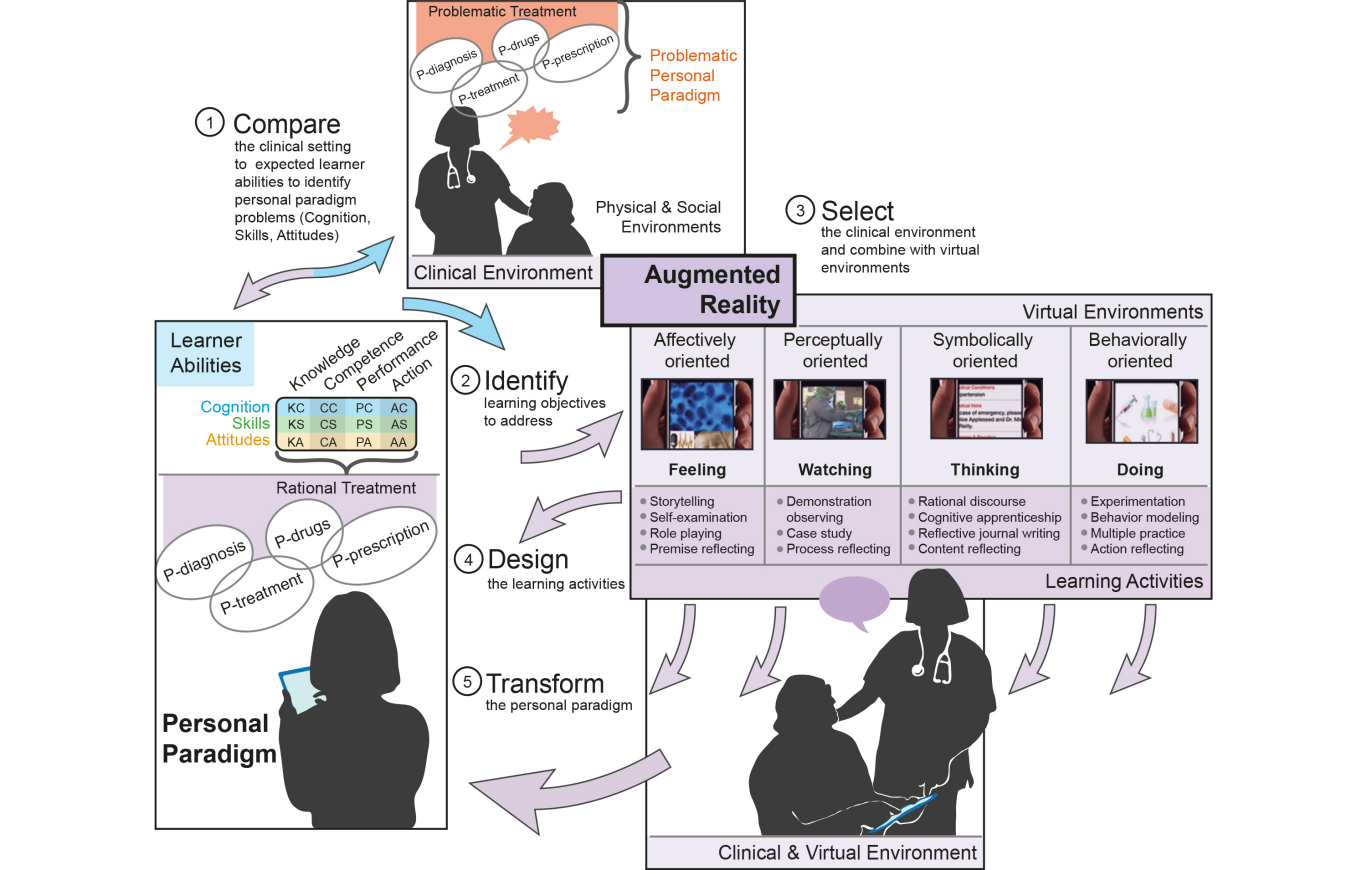
MARE function structure.

We propose the following function structure shown in Figure 3 for developing MARE. The personal paradigm is the starting point of design learning and must transform to become flexible. A physician’s personal paradigm includes his or her personal style of diagnosis, treatment, prescription, and drugs (P-diagnosis, P-treatment, P-prescription and P-drugs, which are four related processes) [13]. The physician’s personal paradigm could be analyzed through observation and deep interviews.

Second, by comparing the learners’ personal paradigms with professional expectations, we can describe the learning objectives and check their problematic reference. The learning activities cycle, which focuses on improving one’s personal paradigm from feeling, watching, and thinking to doing, will help learners reflect on their practice and change the problematic frames of reference.

After identifying the learning objectives, an AR environment of MARE framework should be designed. Four oriented learning environments, which can add different virtual objects to the real clinical environment, create multiple sensory channels for learning [45]. Affective-oriented environments affect health care learners’ feelings. Perception-oriented environments are beneficial for observation. Symbol-oriented environments are particularly useful for thinking, and behavior-oriented environments are beneficial for doing [42].

The real clinical environments are the immediate context in which a connection is needed between learning and practice. The real clinical environment is the anchor and scaffold upon which learners are encouraged to learn. The real clinical environment includes physical environments and social environments. The content in physical environments, such as patients and their disease, microbiological samples, documentation and clinical notes, medical equipment, drugs, and consequences of bacterial resistance, can be the anchor to trigger a learning activity, which then aims to fulfill a learning outcome within the appropriate therapeutic stage. The social environment (ie, local culture and customs, organizational norms, and policy) shapes the content and forms of learning, which should be more instrumental or communicative.

Virtual environments, which are simulated with computers, extend the real-world environment with an assurance of safety and enable or increase opportunities for engagement. Although it may be necessary or attractive for medical learners to learn in the clinical context, observing a real-world context could be dangerous, expensive, or even impossible [46]. Computer-generated content, such as sound, graphics, 3D, video, or text, shows learners an indirect view of surroundings and enhances learners’ different senses to achieve the learning objectives.

In these environments, learning activities are added, which will help medical learners to recognize and build their personal paradigm as they develop skills, gain insights, and determine the dispositions that are essential for translating what they learn into action. Each mixed environment in MARE has its own focus on different learning activities, and the environments should complement and reinforce one another.

#### Personal Paradigm

The personal paradigm is compiled from the frames of reference that shape learners’ beliefs regarding guiding action in transformative learning theory. The personal paradigm combines the individual’s mind-sets, habits, and meaning perspectives, and encompasses cognitive, conative, and affective components. This paradigm is affected by sociolinguistics, moral and ethical values, learning styles, religious beliefs, psychological heath, and aesthetic preferences [43], and is developed through the learners’ learning and/or practice experience. Problematic frames of reference can be caused by poor teaching, disjointed practice, bad example by colleagues, patient pressure, and salesmanship [47].

#### Learning Environment, Assets, and Activities

The learning environment provides the conditions and external stimuli that facilitate learning and transform the learners’ paradigms. Learning assets provide the content for learning [48]. Learning assets are composed of different media forms, such as text, sound, and video; various media can be used in MARE to create different learning environments and realize the valuable functions of different media [49]. MARE mixes real clinical environments and virtual environments in a learning environment within which learners feel, think, watch, and act. Real clinical environments are an immediate context in which learners connect with the learning and practice. These environments include physical environments and social environments. As expected by situation learning theory [39], the clinical environments provide the anchor and scaffold in which learning is encouraged. The virtual environment is useful for learners who learn in different ways and transforms the problematic frames of reference in their personal paradigms. These types of environments conform to create safe environments, in which learners experience learning theories including transformative learning theory [42]. Learning activities are the approach by which learners obtain meaning from learning material, context, and other people in the learning environment. The three learning theories suggest various learning activities, as seen in Table 1. Although an individual’s learning style preferences may be inclined toward specific activities, using diverse learning activities is effective for all learning styles [42].

### Application of Mobile Augmented Reality Education to a Health Care Challenge

In recent years, one of the global health threats has been the spread of antibiotic resistance. Encouraging rational antibiotic use is of paramount concern to authorities worldwide in order to minimize the development of resistance [50]. Multifaceted national and international strategies have been recommended [51]. Education is an important strategy for the rational use of antibiotics. We used the MARE framework to design GP training for the rational use of antibiotics.

Implementing the MARE framework involves several steps: (1) defining the educational outcomes (based on the outcome layer), (2) defining the GP’s personal paradigm, (3) characterizing the learning environment, and (4) designing the learning activities.

### The Outcome Layer of General Practitioners' Rational Use of Antibiotics

#### Overview

The different abilities for rational use of antibiotics were adapted from Public Health England and a number of authors [36-38]. In Tables 1-4, we show how cognition, skill, and attitude can be identified across the spectrum of abilities from knowledge to action. Emotions or attitudes affect the abilities acquired, but do not have a corresponding relationship to specific cognitive and physical skills. We include every affective level in the tables for easy understanding. Attitudes within each level will be surveyed through an attitude questionnaire instrument.

#### Knowledge Level

Knowledge-level expectations for GPs regarding the rational use of antibiotics are shown in Table 1. When GPs use MARE as a tool for evaluating knowledge, they can scan or take a photo of the object in their workplace, task, or virtual case, and the real object will be integrated in MARE. GP behavior can be tracked and their skill within the knowledge level tested. For example, KS1 involves obtaining microbiological cultures or other relevant tests before starting treatment as necessary. A patient who has bacterial pneumonia or viral pneumonia will be shown to a GP treating in MARE. The GP will either select laboratory tests and interpret results or not. We will know whether the GP achieves KS1 or not. The KC of GPs can also be evaluated in MARE. GPs can write instant messages, comment, and annotate that they understand the rational use of antibiotics. GPs can also categorize, tag, or highlight the information that they think is correct. For example, KC2 is recognizing trade and generic names, and the class of prescribed antimicrobials. GPs can categorize the class of prescribed antimicrobials when using MARE to scan trade or generic names.

#### Competence Level

The competence level expected of GPs regarding rational use of antibiotics is described in Table 2. Emotions and values not only affect the application of knowledge but are also a foundation for building GP competence according to physicians’ professional competence definitions [34]. When we use MARE to evaluate GPs’ competence levels, the cases could be conducted in mixed real environments (eg, the real person and the symptom described coexist on the GP’s mobile phone in his or her workplace). The procedure for forecasting, executing, or replying can be uploaded to evaluate the GP's CC and CS. For example, CC4 is constructing a prescription for an antimicrobial with its pharmacokinetics and knowing how this affects the choice of dosage regimen. The case condition will change when different antimicrobials are used with their pharmacokinetics. The result for the forecasting of antibiotics by the GP and the dosage regimen will be evaluated.

#### Performance Level

The performance level expected of GPs regarding the rational use of antibiotics is shown in Table 3. To aid GPs in assessing their workplace performance using the MARE framework, we should build a network for physicians in which they can share their work experiences; then the GPs can review, question, and validate their work performances with each other. Further, the GPs can negotiate, debate, and comment on real cases, and their performance in skills, such as PC and PS, can be tracked and estimated. For example, PS2 is mastering when to use a delayed antimicrobial prescription and how to negotiate this with the patient. One way is to evaluate the GP’s response with the patient case shared on MARE with other GPs meeting at a real clinic. Another way is through creating a story case in which GPs often meet at their workplace to check how the GP deals with delaying antimicrobial prescriptions and negotiating.

#### Action Level

The action level involving the rational use of antibiotics is explained in Table 4. It is hard to evaluate GPs’ real actions, but MARE could be a platform for GPs collaborating, planning, and publishing their views or directing others. As an initiator for action, GPs’ internalized values can regulate the GPs’ pervasive and consistent behavior.

**Figure 4:**
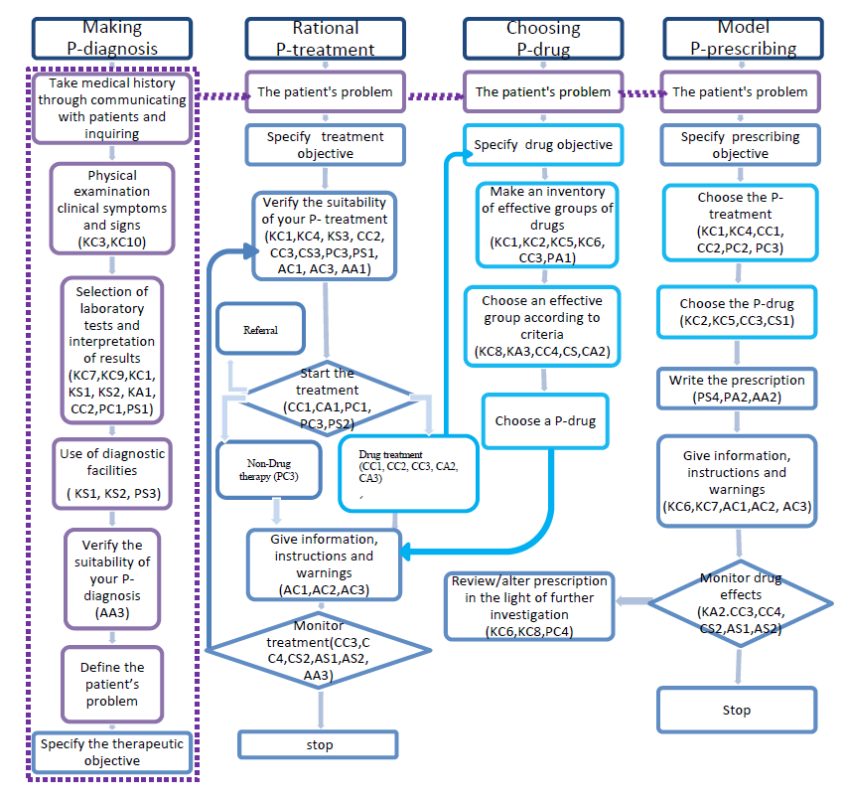
The process of revising the personal paradigm for a rational therapeutic process. The figure content was developed using various sources [13,52,53].

First, we use the expected abilities in Tables 1-4 to analyze the GP’s personal paradigm with the rational therapeutic process (see Figure 4). For example, a GP needs items KC3 and KC10 for physical examination clinical symptoms and signs. Items KC7, KC9, KC10, KS1, and KS2 are the GPs’ abilities when they select laboratory tests and interpret the results, and so on. Each ability item in Figure 4 can be compared with the GP’s current personal paradigm. GPs’ problematic frames of reference for using antibiotics were identified with comparisons. Problematic frames of reference could be caused by a lack of ability or the wrong habit and mind-set. Finding the problem areas will help establish specific learning objectives. Meanwhile, an evaluation tool was developed to assess these specific GP learning outcomes. Content for Figure 4 was developed using various sources [13,52,53].

### General Practitioners' Personal Paradigms About Rational Use of Antibiotics

The GP’s personal paradigm is the means by which he or she sets his or her prescribing behavior for antibiotics. Figure 4 displays the process of revising the personal paradigm for a rational therapeutic process. The components of the GPs’ paradigms with rational use of antibiotics have been described as different abilities in Tables 1-4. The problem of a GP’s paradigm in the real clinical setting could be checked within Figure 4 and Tables 1-4. GPs require different abilities in each phase of the therapeutic process to build their own paradigm with rational treatment as the ultimate aim. Although the P-diagnosis initiates the therapeutic process, each phase in the paradigm could be adapted independently or considered as a whole during the learning process. When a phase is isolated in the independent paradigm for training models, the other relative phases in the paradigms are assumed to be perfect.

In comparison to the expected abilities and the ideal paradigms for a GP’s rational use of antibiotics, GPs will need different abilities in each phase of the therapeutic process. As in Figure 4, we added the expected ability in each stage. For example, GPs need only some KC in a few stages of each phase, and these abilities are the basis of later stages and phases. The ability to combine cognition and skill is needed in most stages, and is shown by being able to progress from knowledge to the performance or action level. Emotions and attitudes are as important to achieving learning objectives as are cognition and skill. As we mentioned before, emotions and attitudes do not map directly to ability level, but rather to the GP paradigm in each stage.

Aside from the abilities that help construct the GP’s personal paradigm, many other factors affect a GP’s paradigm. MARE should help GPs build more accurate personal paradigms or transform problematic frames of reference. In Figure 4, the GP’s existing personal paradigm, the situation, and the characteristics of each stage in the therapeutic process are analyzed. The flow and visualization of relationships can help inform the design of learning activities and learning environments with MARE.

### Learning Environment Design for General Practitioners' Rational Use of Antibiotics

After the learning objectives and the GP’s personal paradigm for the rational use of antibiotics are compared, the learning environment could be designed for GP rational use of antibiotics as follows:

1. In affective-oriented environments, visuals or voice simulations are overlaid in the physical environments to affect the attitudes of GPs in specific settings. GPs are encouraged to share their values and feelings from their concrete experiences.
2. In perception-oriented environments, GPs observe the process simulations of infecting and treating with the real object to reflect and change their habit of misusing of antibiotics. GPs will examine the problem-solving strategies that they used in clinical practice.
3. In symbol-oriented environments, the tasks, guidelines, and alarms are integrated in the therapeutic process to show “the revealed and the concealed” aspects of a complex professional activity. GPs create personal knowledge and develop abilities through discovering, building, and testing hypotheses, and through changing variables and observing the results.
4. In behavior-oriented environments, GPs interact with the virtual object in combination with the real clinical environment to practice what they learn and reflect upon what they do. GPs make their own choices and become more critically reflective to adapt to uncertainty and variable conditions through the decision to act upon a transformed insight.

### Learning Activities Design for General Practitioners' Rational Use of Antibiotics

The learning activities are designed as design strategies for GPs to focus on personal experience during the entire therapeutic process, and to promote reflection on their own personal paradigm in the rational use of antibiotics. The personal paradigm includes four related processes, and correlation and difference functions (as shown in Figure 4), which affect the rational use of antibiotics. In different learning environments, the four types of reflection—premise, process, content, and action—help interpret and give meaning to the GP’s own experience. Within different learning environments, GPs use different learning activities to achieve the learning outcomes for each stage. Table 6 suggests how to apply learning strategies in the four learning environments.

One specific example of the use of MARE as a software app involves examining the effect of AR on emotions and the emotional and cognitive development of physicians within community-based hospitals. Using MARE, we can develop a mobile phone-based software app to be used on the physician’s own mobile phone. GPs who work in community hospitals would be included in the study after they have given informed consent to participate in the trial. During the learning process, the physician participants would take turns role-playing as physicians and patients. As a physician, a GP could see, through his or her mobile phone, the virtual pneumonia infecting a patient via a bacterium or virus. When a GP chooses an antibiotic to treat viral pneumonia or the dose is wrong in the MARE app, the pathogen and commensal change in the patient’s body will appear on the GP’s mobile phone.

**Table 6:** General practitioners' learning activities and application examples in learning environments^a^.

Learning environment	Learning activities	Examples of use in antibiotic education
**Affective oriented**		
	*Role-playing* of GPs^b^ as patients could arouse GPs’ empathy.	GPs can role-play as patients for one another and use MARE^c^ tracking to experience how patients may feel or change during the treatment process.
	*Storytelling* could be used to share GPs’ experiences to become aware of their own problems.	GPs will be encouraged to tell stories related to the situation being addressed by MARE or add as new cases within MARE.
	*Self-examination* or discussion with peers could raise consciousness about the rational use of antibiotics.	After learning with MARE, GPs examine or discuss with peers how they feel about the learning experience.
	*Premise reflecting* may lead to transforming the GPs’ belief systems in the use of antibiotics.	GPs assess assumptions about what determines or guides prescribing antibiotics within their value systems. Disorienting dilemmas should be designed to define problem processes that provide an opportunity for GPs to reflect on MARE.
**Perception oriented**		
	*Demonstration observing* could provide GPs the right therapeutic skills and transformed insights regarding infectious diseases.	GPs can observe antimicrobial therapy dynamic change processes, which simulate a demonstration of the complex interrelationship between patient, microorganisms, and antimicrobial drugs through MARE.
	*Case studies* could improve the GPs with the ability to analyze and resolve problems.	GPs can analyze well-designed case descriptions of misused antibiotics on MARE and offer solutions and recommendations related to a concrete situation or problem they might meet in the real clinical environment.
	*Process reflecting* questions the etiology and factors of actions that might change GPs’ problem-solving strategies during the therapeutic process.	GPs compare their own problem-solving process with expert modeling or others in MARE to examine their strategies for appropriate use of antibiotics.
**Symbol oriented**		
	*Cognitive apprenticeship*, which makes thinking visible, could iteratively build the GPs’ intellectual skills in rational use of antibiotics.	GPs follows the guidelines, posters, or cue cards for the rational use of antibiotics in MARE to build their cognitive ability, as described in Tables 1-4.
	*Rational discourse* could offer GPs accurate and complete information with which to get objective and rational consensus on the rational use of antibiotics.	GPs have an equal opportunity to participate in a rational discourse with a challenging incident or controversial statement about the use of antibiotics, which was designed in MARE.
	*Reflective journal writing* could help GPs externalize and articulate what they are learning.	GPs write a blog with MARE to record their ideas, thoughts, and feelings about the events they have observed, in order to learn and gain experience.
	*Content reflecting* involves GPs thinking back to what was done in past experiences, which might transform their meaning scheme for the use of antibiotics.	GPs examine past experiences of their personal paradigms with the use of antibiotics and compare them to the content of guidelines for the rational use of antibiotics and/or engage in comparative discourse with others through MARE.
**Behavior oriented**		
	*Experimenting* through scientific-based inquiry methods for problem solving could help GPs develop critical thinking and adapt to changing contexts and new challenges.	GPs use MARE in the experimentation mode, which illustrates phenomena and variables of typical cases involving the use of antibiotics, in order to test their ideas, gather data, and distill the results.
	*Behavior modeling* could engage GPs in practicing their skills for the desired behavior of the rational use of antibiotics.	GPs participate in interactions with the model, which simulates the desired behavior for the rational use of antibiotics on MARE to help GPs practice and master their skills.
	*Multiple practices* could enhance a GP’s rational use of antibiotics behavior.	GPs participate in planned exercises that use scaffolds and combine different real conditions to practice what the GPs learn in MARE.
	*Action reflecting* could help GPs apply their current experiences of solving problems in clinical practice to future problems.	GPs think about the problem and the solutions involving the use of antibiotics when they meet in practice. This reflection-in-action or reflection-on-action could be reinforced with MARE.

^a^The table content was developed using various sources [54-56].

^b^General practioners (GPs).

^c^Mobile augmented reality education (MARE).

## Discussion

### Principal Findings

AR, as well as many other information technologies, is expected to help the reform of health professional learning. AR is a promising technology that can amend curricula rigidity, traditional pedagogy, and adaptation to local contexts in health education [57]. AR has shown its potential as a learning technology in health care education, but most AR systems are still used in traditional pedagogies [4], just like online learning did when it was first introduced.

Effective application of technology in education practice requires profound understanding of the potential of technologies and the specific disciplines, as well as appropriate learning theory support [58]. The MARE framework through a CFAM considers the characteristics of AR and the learning theory supporting it, as well as the objective identification to guide design. This full view of MARE is helpful for medical education to improve professional development from knowledge to practice. The three learning theories provide foundational support from the different views of the relationship among learning, practice, and environment. The outcome layer, which analyzes different ability levels from knowledge to practice, can possibly avoid “teaching pitched at the wrong level” [30], and it can also fill the gap between teaching and clinical practice needs. Moreover, AR is a potential tool to help health care educators fill the gap between teaching and clinical practice, especially through guidance by theories to achieve the aim. The MARE framework meets clinical teaching goals listed in the Association for Medical Education in Europe (AMEE) Guides that apply relevant educational theories to guide clinical teaching in the hospital setting [29].

### Limitations

This is the first AR framework based on learning theory with clear objectives for guiding the design, development, and application of mobile AR in medical education. To date, there is no standard methodology for designing an AR framework. MARE uses a CFAM, which is based on a theory that provides systematic understanding of the multidisciplinary, complex relationship from knowledge to practice in medical education. However, this MARE framework created through a CFAM from multidisciplinary publications and reference materials must be tested in practice.

Validation of the framework was suggested by Jabareen [24], but he did not give a method for how to validate it. We checked the internal validity by involving authors from different disciplines and perspectives to reduce the bias. We also used this framework for analysis of, and application in, GPs’ rational use of antibiotics. However, since this is a general framework for guiding the design, development, and application of AR in medical education, external validity, which is transferable in qualitative research, must be further tested with users and with the next step to develop an AR app. In addition, a number of experts such as instructional designers, AR developers, GPs, medical educators, visual designers, information and communications technology (ICT) specialists, and interaction designers are needed to further design, improve, and test the framework.

### Comparison With Prior Work

The MARE framework is a general instructional design framework that addresses functional conceptualism by explaining and predicting theory with a multidisciplinary perspective [6,59]. Similarly, the general instructional design framework has been used to design e-learning and simulation training frameworks. Situation learning theory was used to guide the design of the learning environment and learning activities for an instructional design model, and transformative learning theory was used to build an e-learning framework [54]. Identifying the learning aim is important for a framework that uses the design process in electrical engineering as a model [60]. Edelson developed a framework with principles and learning activities from the inquiry-based cycle [61]. Distinct from these frameworks, the MARE framework tries to meet all components of functional conceptualism: goal, values, functions, and situations. Learning theories are the foundation of the MARE supporting values. Their selection corresponds to the characteristics of AR and GP learning outcomes. Clarifying the learning goal is the important first step in MARE instructional design. Learning activities are manipulable variables within learning environments. Activities are suggested from learning theories to achieve learning outcomes. Learning activities are described along with the situations for guiding when and how to apply them in the MARE framework.

### Implications and Future Work

The proposed MARE design framework addresses the lack of theory for guiding the design, development, and application of AR to improve GPs’ rational use of antibiotics. Understanding the theory behind this framework could benefit instructional designers, AR developers, and GP professionals when they apply the recommendations and could ultimately lead to further development of this framework and its practical use.

The first implication of MARE for AR designers is how to apply learning theories and learning outcomes to guide AR instructional design. Situated learning theory, experiential learning theory, and transformative learning theory share some views, but each has unique emphases. The learning activities from which the learning theories are based are effective substitutes for traditional medical instruction in AR environments. The fundamental change in pedagogical philosophy is better than the tinkering with “interactivity” levels by instructional designers to support deeper, richer levels of learning [54]. The learning outcome framework (Figure 2), which combines Miller’s pyramid of clinical assessment and Bloom’s taxonomy of learning aims, avoids assessment that rests on low ability. AR designers may use the learning outcomes, which are explained in Tables 1-4, to analyze a GP’s personal paradigm and to design their AR program. The effectiveness of the strategies and the appropriateness of the goals require further evaluation and refinement.

The second implication of MARE for an AR developer is the function framework. It may help developers understand how to create mixed environments for learning, not just for technology-driven infotainment. Different environments offer different learning functions. AR developers may use the list of teaching activities shown with the MARE framework as guidance when they consider how to develop AR functions. In terms of the learning objective, learning environment, learning activities, GP personal paradigm, and therapeutic process, AR developers may think about how to build interactive models and interactive levels between MARE and GPs in different environments. The learning materials in different environments must be designed and developed.

Another implication of MARE for GP educators and researchers is the new technology and learning activity supported by learning theory, which corresponds to technology characters. GP educators and researchers may integrate it in their instructional practice. They can use the list of broader opportunities of MARE outcomes to compare with their students’ learning needs to design an app. The framework could be used to guide other drug or therapeutic intervention education.

### Conclusions

Due to the traditional teaching focus on recalling facts, health care professionals face the challenge of transforming knowledge into practice in health care settings. AR could provide a means to resolve this challenge, but it lacked a theory-guided design. Most AR apps still use traditional learning activities—see one, do one, teach one—in medical education, which hinders its educational function.

This paper has described a framework for guiding the design, development, and application of MARE to health care education. This includes consideration of a foundation, a function, and a series of outcomes. The foundation based upon three learning theories enhances the relationship between practice and learning. The function constituted by suggested learning activities and the requirements of the learning environment from the foundation and AR characteristics can amend the gap in the learning outcomes and medical learners’ personal paradigms. The learning outcome, which combines Miller’s pyramid and Bloom’s taxonomy, can clarify the objectives and expectations and avoid teaching pitched at the wrong level [29].

Furthermore, we used a global health challenge—antibiotic resistance—as an application example and chose one important aspect that is the general practitioners’ rational use of antibiotics, to which to apply the MARE framework. With this framework, the expected abilities of GPs’ rational use of antibiotics are described specifically and may easily be executed and evaluated. The abilities were compared with the GP personal paradigm to solidify GP practical learning objectives and to help design learning environments and activities. Future work will focus on the implementation of the proposed framework by developing a mobile phone-based AR app for GP training and for conducting evaluations in China.

## Abbreviations

AC: cognition within the action level
AMEE: Association for Medical Education in Europe
AR: augmented reality
AS: skill within the action level
CC: cognition within the competency level
CFAM: conceptual framework analysis method
CINAHL: Cumulative Index to Nursing and Allied Health Literature
CS: skill within the competency level
ERIC: Education Resources Information Center
GP: general practitioner
GPS: global positioning system
ICT: information and communications technology
ISO: International Organization for Standardization
KC: cognition within the knowledge level
KS: skill within the knowledge level
MARE: mobile augmented reality education
P-diagnosis: personal style of diagnosis
P-drugs: personal style of drugs
P-prescription: personal style of prescription
P-treatment: personal style of treatment
PC: cognition within the performance level
PS: skill within the performance level
